# The role of adenocarcinoma subtypes and immunohistochemistry in predicting lymph node metastasis in early invasive lung adenocarcinoma

**DOI:** 10.1186/s12885-024-11843-4

**Published:** 2024-01-29

**Authors:** Mengchao Xue, Junjie Liu, Zhenyi Li, Ming Lu, Huiying Zhang, Wen Liu, Hui Tian

**Affiliations:** Department of Thoracic Surgery, Qilu Hospital, Shandong University, Lixia District, Jinan City, Shandong Province China

**Keywords:** Invasive lung adenocarcinoma, Lymph node metastasis, Predictive models, Nomogram

## Abstract

**Background:**

Identifying lymph node metastasis areas during surgery for early invasive lung adenocarcinoma remains challenging. The aim of this study was to develop a nomogram mathematical model before the end of surgery for predicting lymph node metastasis in patients with early invasive lung adenocarcinoma.

**Methods:**

In this study, we included patients with invasive lung adenocarcinoma measuring ≤ 2 cm who underwent pulmonary resection with definite pathology at Qilu Hospital of Shandong University from January 2020 to January 2022. Preoperative biomarker results, clinical features, and computed tomography characteristics were collected. The enrolled patients were randomized into a training cohort and a validation cohort in a 7:3 ratio. The training cohort was used to construct the predictive model, while the validation cohort was used to test the model independently. Univariate and multivariate logistic regression analyses were performed to identify independent risk factors. The prediction model and nomogram were established based on the independent risk factors. Recipient operating characteristic (ROC) curves were used to assess the discrimination ability of the model. Calibration capability was assessed using the Hosmer–Lemeshow test and calibration curves. The clinical utility of the nomogram was assessed using decision curve analysis (DCA).

**Results:**

The overall incidence of lymph node metastasis was 13.23% (61/461). Six indicators were finally determined to be independently associated with lymph node metastasis. These six indicators were: age (*P* < 0.001), serum amyloid (SA) (*P* = 0.008); carcinoma antigen 125 (CA125) (*P* = 0. 042); mucus composition (*P* = 0.003); novel aspartic proteinase of the pepsin family A (Napsin A) (*P* = 0.007); and cytokeratin 5/6 (CK5/6) (*P* = 0.042). The area under the ROC curve (AUC) was 0.843 (95% CI: 0.779–0.908) in the training cohort and 0.838 (95% CI: 0.748–0.927) in the validation cohort. the *P*-value of the Hosmer–Lemeshow test was 0.0613 in the training cohort and 0.8628 in the validation cohort. the bias of the training cohort corrected C-index was 0.8444 and the bias-corrected C-index for the validation cohort was 0.8375. demonstrating that the prediction model has good discriminative power and good calibration.

**Conclusions:**

The column line graphs created showed excellent discrimination and calibration to predict lymph node status in patients with ≤ 2 cm invasive lung adenocarcinoma. In addition, the predictive model has predictive potential before the end of surgery and can inform clinical decision making.

**Supplementary Information:**

The online version contains supplementary material available at 10.1186/s12885-024-11843-4.

## Introduction

Lung cancer (LC) is the second most prevalent tumor and remains the leading cause of malignancy-related deaths worldwide by far [1]. LC is commonly classified into small cell lung cancer (SCLC) and non-small cell lung cancer (NSCLC). Among them, adenocarcinoma is the most important subtype of NSCLC and the most common type of LC. With the increasing popularity of low-dose spiral computed tomography (CT) in health screening and disease diagnosis, the incidence of ≤ 2 cm lung cancer has been increasing [2]. For early-stage lung adenocarcinoma, more thoracic surgeons are accepting segmental or subsegmental resection and selective lymph node dissection as the optimal treatment modality [3, 4]. However, in some LC cases, lymph node metastasis (LNM) occurs in the early stages of the tumor. The incidence of LNM in LC cases with lesions ≤ 2 cm in diameter has been reported to be about 10% [5, 6]. Emerging evidence suggests that lymph node metastasis is a risk factor for poor prognosis in patients with early-stage lung adenocarcinoma [7]. Unfortunately, the accuracy of preoperative lymph node staging CT scans is only 45%-79% [8–12]. Preoperative mediastinoscopy and endobronchial ultrasound transbronchial needle aspiration are not routinely used in patients with clinical stage I disease, and these methods have produced a considerable number of false-negative results [13–15]. Complete clearance of metastatic lymph nodes during surgery plays a key role in improving the disease-free survival and overall survival of patients [16]. Therefore, it is necessary to accurately assess preoperative lymph nodes metastasis in NSCLC.

It has been shown that adenocarcinomas with micropapillary and solid growth patterns are more aggressive and have a poorer prognosis [17, 18]. In addition, blood inflammatory markers and tumor markers can be used to predict lymph node metastasis in lung cancer [19–22]. CT remains the most widely used tool to assess tumor and lymph node involvement in patients with early-stage non-small cell lung cancer [8–11]. Some researchers claim that frozen sections are a key indicator to guide the approach to resection [23] and that it is feasible to report histological subtypes and other pathological features during surgery [24, 25].

To date, many studies have explored independent predictors of lymph node metastasis [26–32]. These include carcinoembryonic antigen (CEA) [26], tumor size [26], standardized uptake value maximum (SUVmax) [27], female [28], never smoker[28], adenocarcinoma histology [28], positive N1 lymph nodes on positron emission tomography (PET) [29], blood inflammation biomarkers [30], neutrophil to lymphocyte ratio (NLR) [31] and consolidation-to-tumor ratio (CTR) [32], ect. However, only a few studies have developed comprehensive models to predict lymph node metastasis based on radiological features, patient clinical information, and hematological parameters.

In our study, we explored the risk factors for lymph node metastasis in a cohort of patients with early invasive lung cancer and developed a nomogram model for predicting the risk of lymph node metastasis based on patient clinical information, hematologic indicators, imaging features, and pathologic findings. The aim was to enable the nomogram to quickly and accurately predict the incidence of lymph node metastasis before or during surgery, which may provide a computational method for surgeons to make intraoperative decisions.

## Materials and methods

### Patients

This study was approved by the Ethics Committee of Qilu Hospital, Shandong University (registration number: KYLL-202008–023-1), and all patients signed an informed consent form for the use of their clinical information prior to the procedure.

Patients with invasive adenocarcinoma from January 2020 to December 2021 at Qilu Hospital of Shandong University were retrospectively evaluated.

The inclusion criteria were: (1) patients with a single intrapulmonary nodule suggested by chest CT within 1 month before surgery; (2) nodules with a maximum diameter ≤ 20 mm on CT; (3) undergoing pneumonectomy (lobectomy or subpneumonectomy) with systemic lymph node dissection; (4) complete pathological data and pathological type of Invasive lung adenocarcinoma; (5) not receiving neoadjuvant chemotherapy or radiotherapy before surgery; (6) no pulmonary atelectasis and active inflammatory images of the lungs. Exclusion criteria were (1) patients < 18 years of age, (2) open-heart surgery, (3) incomplete perioperative data, and (4) patients with a history of malignant disease within 5 years. (5) combination of acute infectious diseases that can cause changes in the levels of systemic inflammatory markers; (6) presence of distant metastases.

**Fig. 1:**
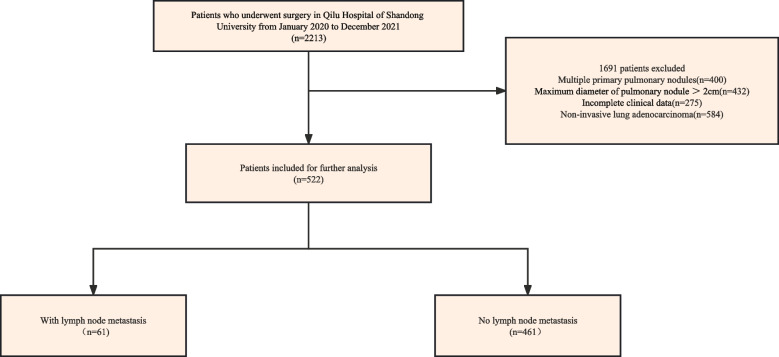
Flow chart of this study

A total of 2213 patients were included in this study, and after our exclusion according to the above-mentioned criteria, 522 patients with invasive lung adenocarcinoma with tumor size ≤ 2 cm were finally recruited in our study. Figure 1 shows the flow chart of included patients.

### Clinical data of patients

Clinicopathological information was collected from the patient record management system as follows: age, gender, presence of preoperative comorbidities [hypertension, diabetes mellitus, and chronic obstructive pulmonary disease (COPD)], history of smoking, body mass index (BMI), predicted percent forceful expiratory volume in one second (FEV1% predicted), predicted percent maximum voluntary ventilation (MVV% predicted), and American Society of Anesthesiologists (ASA) score.

### Hematological test

Record hematologic parameters within 2 weeks prior to surgery as follows. (1) Blood count: neutrophils, basophils, eosinophils, lymphocytes, monocytes, red blood cells, platelets, albumin, hemoglobin, blood glucose, blood type. (2) Serum enzyme count: serum 5'-nucleotidase (5'-NT), serum amylase (SA), lactate dehydrogenase (LDH). (3) Tumor markers: carcinoembryonic antigen 125 (CA125), neuron-specific enolase (NSE), carcinoembryonic antigen (CEA), gastrin-releasing peptide (pro-GRP), cytokeratin 19-fragment (cybra21-1), and squamous carcinoma antigen (SCC). (4) Inflammatory markers: serum complement C1q and derived neutrophil–lymphocyte ratio (NLR), platelet-lymphocyte ratio (PLR), monocyte-lymphocyte ratio (MLR), derived neutrophil–lymphocyte ratio (dNLR), neutrophil–lymphocyte and platelet ratio (NLPR), systemic inflammatory response syndrome (SIRS), total systemic inflammatory index (AISI) and systemic inflammatory index (SII). These derived inflammatory indicators were calculated as follows.

- NLR = neutrophils/lymphocytes.
- PLR = platelets/lymphocytes.
- MLR = monocytes/lymphocytes.
- dNLR = [neutrophils/ (leukocytes—neutrophils)].
- NLPR = [Neutrophils/ (lymphocytes × platelets)].
- SIRI = [(neutrophils × monocytes)/lymphocytes)].
- AISI = [(neutrophils × monocytes × platelets)/lymphocytes].
- SII = [(neutrophils x platelets)/lymphocytes)].

### Imaging analysis

The morphological features of computed tomography include: location (central or peripheral), shape (regular or irregular), spiculation, calcification, cavity sign, bronchial sign, lobar sign, pleural adhesion sign, vascular penetration sign, pleural effusion sign, maximum tumor diameter, lymph node enlargement sign, and consolidation to tumor ratio (CTR). Two radiologists measured each imaging feature independently, and a third radiologist with more than 20 years of experience in chest radiology reassessed the discrepancies. Any disagreements were resolved by consensus.

Centrality was defined as nodules located in the bronchi, lobular bronchi, and segmental bronchi. Peripherality was defined as nodules located below the tertiary bronchi. Spiculation was defined as spread from the nodal margins to the lung parenchyma without contacting the pleural surface. Signs of calcification were defined as having one of these patterns on CT imaging: stratification, central nodule, diffusion, or popcorn pattern. Cavitation signs were defined as gas-filled spaces that are considered to be transparent or low-attenuation regions. The bronchial sign shows direct bronchial involvement of nodules on CT images. Lobulation was defined as the wavy or fan-shaped portion of the lesion surface and the strands extending from the nodal margins into the lung parenchyma. Signs of pleural adhesions were defined as linear attenuation or major or minor fissures toward the pleura. The vascular penetration sign was observed on the CT image with a pulmonary artery crossing the node. The pleural effusion sign was defined as a blunting of the rib-diaphragm angle visible on the CT image. The lymph node enlargement sign was the enlargement of mediastinal lymph nodes that can be observed on CT images.CTR was defined as the ratio of the diameter of the solid component of the lung nodule to the maximum diameter of the nodule.

### Histological evaluation

All pathological specimens were fixed in formalin, stained with hematoxylin–eosin, and evaluated by two experienced lung pathologists. Histopathological evaluation was performed by examining hematoxylin–eosin-stained slides with a light microscope. All specimens were classified according to the International Association for the Study of Lung Cancer/American Thoracic Society/European Respiratory Society classification of adenocarcinoma of the lung [33]. The pathological lymph node status of patients was confirmed according to the 8th edition of the TNM lung cancer classification.

The percentage of each histological component (mucinous, lepidic, acinar, papillary, micropapillary and solid pattern) was recorded in 5% increments and the tumors were classified according to the predominant pattern. The pattern was considered present if ≥ 5% of the histological pattern was present in the tumor.

### DNA purification and quantification

Cutting all formalin-fixed paraffin-embedded (FFPE) specimens to 5–8 μm thickness. Thereafter, DNA and RNA extraction was performed using 5–30 tissue sections with at least 2% tumor cells using the FFPE DNA/RNA Nucleic Acid Extraction Kit (No. 8.0223601X036G, Xiamen Diagnostics, Xiamen, China). After isolation of DNA and RNA, the concentrations of DNA and RNA were determined using a microscopic spectrophotometer. the RNA concentrations ranged from 10 to 500 ng/μL and the DNA concentrations were > 2 ng/μL.

### Immunohistochemistry Validation in Resected Patients

All IHC staining was performed in the clinical immunohistochemistry laboratory of our hospital pathology department. All IHC staining was performed in the clinical immunohistochemistry laboratory of our hospital pathology department. Briefly, specimens were sectioned at 5 μm, dewaxed and incubated with primary antibody. Staining characteristics as well as the intensity and distribution of staining patterns were reviewed and considered. If more than 5% of the tumor cells with the appropriate staining pattern were found, the case was considered positive; otherwise, the case was considered negative. Immunohistochemistry was verified for CK5/6, CK7, Napsin A, MUC-AC, P63, Ki-67% positive rate, CyclinD1, EMA, CD31, D2-40, etc.

### special staining in resected patients

The Periodic Acid-Schiff (PAS) reaction, Periodic Acid-Schiff reaction with diastase (PAS-D) and elastic fibers are three special staining procedures that are commonly performed in a histology laboratory. The staining reaction was classified as positive or negative by three "blinded" observers.

### *Statistical* analysis

All statistical analyses were performed using SPSS 26.0 (SPSS Inc., Chicago, Illinois, USA) and R statistical software (Windows version 4.2.1, http: //www.r-project.org/). We used the “rms package” to plot the nomogram, “pROC” to plot the ROC curve, and “rmda” to plot the DCA curve. Categorical variables were compared using Pearson's Chi-square test or Fisher's exact test. Normally distributed continuous variables were expressed as mean ± standard deviation (SD) and compared using the Student's t-test. For non-normally distributed continuous variables, data were expressed as medians (interquartile range [IQR]) and compared between two groups using the Mann–Whitney U test. Statistical significance was described as a two-sided P value of less than 0.05.

We implement the random assignment of patients through the R. All enrolled patients were randomly assigned to the training and validation cohorts in a 7:3 ratio, using a randomly segmented sample. The training cohort was used to develop the prediction nomograms, while the validation cohort was used to verify the performance of the nomograms.

### Predictive model development and validation

#### Construction of nomogram

The training cohort data were first analyzed by univariate logistic regression analysis to identify potential risk factors. Those factors with P-values less than 0.05 in univariate analysis were included in further multivariate logistic regression analyses. Finally, predictive models were developed using independent risk factors (*P* < 0.05 in multivariate logistic regression). A nomogram was created by using R statistical software (Windows version 4.2.1, http: //www.r-project.org/). Area under the curve (AUC) was determined, and receiver operating characteristic (ROC) curves were created. A regression model was used to calculate scores for each variable, and the predicted probability of risk of lymph node metastasis in small-sized non-small cell lung cancer could be derived by summing the scores for each variable.

### Nomogram performance

An assessment of the performance of predictive nomograms is made by discriminative power, calibration and clinical utility. Discriminative power is the capability of a model to correctly differentiate between events and non-events.ROC curves are employed to assess the recognition efficiency of predictive nomograms [34]. A measurement of how well the predicted probability matches the actual result is called calibration. the Hosmer–Lemeshow test can be used to assess calibration ability, with a *p*-value greater than 0.05 indicating satisfactory calibration [35]. Subsequently a nomogram calibration plot is formed to further assess the calibration. This was verified internally by using a bootstrap method repeated 1000 times [36]. Predictive nomograms were evaluated for clinical effectiveness using decision curve analysis (DCA) based on the net benefit of different threshold probabilities [37]. The optimal cutoff value was determined when the Youden index (sensitivity + specificity-1) reached its maximum value based on ROC curve analysis of the training cohort.

## Results

### Patient characteristics

A total of 522 patients were enrolled in this study. The overall incidence of lymph node metastasis was 13.23% (61/461). Of all patients enrolled, 284 were women and 138 were men. The median age was 61 (range: 31–81) years. the median tumor size on CT was 1.2 (range: 0.3–2) cm. Demographic characteristics and variable data for both cohorts are shown in Table 1. The training cohort included 366 (70.1%) patients, whereas the validation cohort included 156 (29.9%) patients. The characteristics of the two cohorts were similar, with *p*-values > 0.05 except for MVV% predicted, and the differences in distribution were not statistically significant. Detailed information on the features of the two groups in the training and validation groups is shown in Table 2.

**Table 1:** Patients’ characteristics of the training cohort and validation cohort

Characteristics	All cohort (*N* = 522)	Validation cohort (*N* = 156)	Training cohort (*N* = 366)	*p*
Gender, n (%)				0.171
Female	284 (54.4)	92 (59.0)	192 (52.5)	
Male	238 (45.6)	64 (41.0)	174 (47.5)	
Hypertension, n (%)				0.713
No	352 (67.4)	107 (68.6)	245 (66.9)	
Yes	170 (32.6)	49 (31.4)	121 (33.1)	
Diabetes, n (%)				0.296
No	454 (87.0)	132 (84.6)	322 (88.0)	
Yes	68 (13.0)	24 (15.4)	44 (12.0)	
COPD, n (%)				0.279
No	516 (98.9)	153 (98.1)	363 (99.2)	
Yes	6 (1.1)	3 (1.9)	3 (0.8)	
Smoking history, n (%)				0.338
Non-smoker	373 (71.5)	116 (74.4)	257 (70.2)	
Smoker	149 (28.5)	40 (25.6)	109 (29.8)	
Blood type, n (%)				0.661
A	150 (28.7)	44 (28.2)	106 (29.0)	
B	191 (36.6)	52 (33.3)	139 (38.0)	
AB	58 (11.1)	19 (12.2)	39 (10.7)	
O	123 (23.6)	41 (26.3)	82 (22.4)	
ASA, n (%)				0.859
1	41 (7.9)	11 (7.1)	30 (8.2)	
2	460 (88.1)	138 (88.5)	322 (88.0)	
3	21 (4.0)	7 (4.5)	14 (3.8)	
Location, n (%)				0.714
Centrality	61 (11.7)	17 (10.9)	44 (12.0)	
Peripherality	461 (88.3)	139 (89.1)	322 (88.0)	
Shape, n (%)				0.584
Regularity	175 (33.5)	55 (35.3)	120 (32.8)	
Irregularity	347 (66.5)	101 (64.7)	246 (67.2)	
Spiculation, n (%)				0.77
No	162 (31.0)	47 (30.1)	115 (31.4)	
Yes	360 (69.0)	109 (69.9)	251 (68.6)	
Cavitation sign, n (%)				0.333
No	412 (78.9)	119 (76.3)	293 (80.1)	
Yes	110 (21.1)	37 (23.7)	73 (19.9)	
Calcification, n (%)				0.142
No	517 (99.0)	156 (100.0)	361 (98.6)	
Yes	5 (1.0)	0 (0.0)	5 (1.4)	
Vascular penetration sign, n (%)				0.802
No	140 (26.8)	43 (27.6)	97 (26.5)	
Yes	382 (73.2)	113 (72.4)	269 (73.5)	
Pleural adhesions, n (%)				0.723
No	190 (36.4)	55 (35.3)	135 (36.9)	
Yes	332 (63.6)	101 (64.7)	231 (63.1)	
Bronchus sign, n (%)				0.514
No	352 (67.4)	102 (65.4)	250 (68.3)	
Yes	170 (32.6)	54 (34.6)	116 (31.7)	
Lobulation, n (%)				0.2
No	262 (50.2)	85 (54.5)	177 (48.4)	
Yes	260 (49.8)	71 (45.5)	189 (51.6)	
Lymph node enlargement sign, n (%)				0.677
No	426 (81.6)	129 (82.7)	297 (81.1)	
Yes	96 (18.4)	27 (17.3)	69 (18.9)	
Pleural effusion sign, n (%)				0.853
No	516 (98.9)	154 (98.7)	362 (98.9)	
Yes	6 (1.1)	2 (1.3)	4 (1.1)	
Lepidic, n (%)				0.362
No	162 (31.0)	44 (28.2)	118 (32.2)	
Yes	360 (69.0)	112 (71.8)	248 (67.8)	
Acinar, n (%)				0.141
No	97 (18.6)	23 (14.7)	74 (20.2)	
Yes	425 (81.4)	133 (85.3)	292 (79.8)	
Papillary, n (%)				0.453
No	314 (60.2)	90 (57.7)	224 (61.2)	
Yes	208 (39.8)	66 (42.3)	142 (38.8)	
Micropapillary, n (%)				0.495
No	421 (80.7)	123 (78.8)	298 (81.4)	
Yes	101 (19.3)	33 (21.2)	68 (18.6)	
Solid, n (%)				0.862
No	490 (93.9)	146 (93.6)	344 (94.0)	
Yes	32 (6.1)	10 (6.4)	22 (6.0)	
Mucinous, n (%)				0.642
No	446 (85.4)	135 (86.5)	311 (85.0)	
Yes	76 (14.6)	21 (13.5)	55 (15.0)	
CK5/6, n (%)				0.098
No	496 (95.0)	152 (97.4)	344 (94.0)	
Yes	26 (5.0)	4 (2.6)	22 (6.0)	
CK7, n (%)				0.921
No	393 (75.3)	117 (75.0)	276 (75.4)	
Yes	129 (24.7)	39 (25.0)	90 (24.6)	
TTF-1, n (%)				0.746
No	373 (71.5)	113 (72.4)	260 (71.0)	
Yes	149 (28.5)	43 (27.6)	106 (29.0)	
Napsin A, n (%)				0.154
No	452 (86.6)	130 (83.3)	322 (88.0)	
Yes	70 (13.4)	26 (16.7)	44 (12.0)	
MUC-AC, n (%)				0.064
No	494 (94.6)	152 (97.4)	342 (93.4)	
Yes	28 (5.4)	4 (2.6)	24 (6.6)	
P63, n (%)				0.184
No	483 (92.5)	148 (94.9)	335 (91.5)	
Yes	39 (7.5)	8 (5.1)	31 (8.5)	
CyclinD1, n (%)				0.07
No	493 (94.4)	143 (91.7)	350 (95.6)	
Yes	29 (5.6)	13 (8.3)	16 (4.4)	
EMA, n (%)				0.156
No	496 (95.0)	145 (92.9)	351 (95.9)	
Yes	26 (5.0)	11 (7.1)	15 (4.1)	
CD31, n (%)				0.268
No	491 (94.1)	144 (92.3)	347 (94.8)	
Yes	31 (5.9)	12 (7.7)	19 (5.2)	
D2-40, n (%)				0.403
No	492 (94.3)	145 (92.9)	347 (94.8)	
Yes	30 (5.7)	11 (7.1)	19 (5.2)	
Stretch fiber, n (%)				0.893
No	376 (72.0)	113 (72.4)	263 (71.9)	
Yes	146 (28.0)	43 (27.6)	103 (28.1)	
PAS, n (%)				0.82
No	466 (89.3)	140 (89.7)	326 (89.1)	
Yes	56 (10.7)	16 (10.3)	40 (10.9)	
PAS-D, n (%)				0.828
No	474 (90.8)	141 (90.4)	333 (91.0)	
Yes	48 (9.2)	15 (9.6)	33 (9.0)	
Albumin (g/L), median (IQR)	60.00 (57.92, 62.20)	59.45 (57.68, 61.73)	60.20 (58.02, 62.30)	0.051
Lymphocyte (× 109/L), median (IQR)	1.77 (1.44, 2.19)	1.78 (1.42, 2.21)	1.77 (1.45, 2.19)	0.843
PNI (%), median (IQR)	69.15 (66.00, 71.85)	68.80 (65.84, 71.20)	69.32 (66.11, 72.04)	0.138
Neutrophil (× 109/L), median (IQR)	3.00 (2.46, 3.89)	3.06 (2.49, 3.87)	2.96 (2.45, 3.89)	0.813
Eosinophil (× 109/L), median (IQR)	0.11 (0.06, 0.19)	0.11 (0.07, 0.18)	0.11 (0.06, 0.19)	0.839
Basophil (× 109/L), median (IQR)	0.03 (0.02, 0.04)	0.03 (0.02, 0.04)	0.03 (0.02, 0.04)	0.89
Monocyte (× 109/L), median (IQR)	0.42 (0.34, 0.51)	0.42 (0.33, 0.50)	0.42 (0.34, 0.51)	0.718
Erythrocyte (× 1012/L), median (IQR)	4.50 (4.20, 4.83)	4.49 (4.11, 4.86)	4.50 (4.23, 4.82)	0.383
Hemoglobin (g/L), median (IQR)	138.00 (128.00, 148.00)	137.00 (126.00, 146.00)	138.50 (129.00, 149.00)	0.133
Platelet (× 109/L), median (IQR)	234.00 (198.25, 267.00)	232.00 (194.25, 264.00)	235.00 (199.00, 269.00)	0.319
NLR (%), median (IQR)	1.72 (1.29, 2.24)	1.75 (1.30, 2.27)	1.71 (1.29, 2.23)	0.928
PLR (%), median (IQR)	132.06 (103.89, 163.59)	130.94 (97.47, 164.23)	133.94 (105.04, 163.06)	0.345
MLR (%), median (IQR)	0.23 (0.19, 0.29)	0.22 (0.18, 0.29)	0.23 (0.19, 0.29)	0.438
dNLR (%), median (IQR)	1.28 (1.01, 1.59)	1.28 (0.99, 1.62)	1.28 (1.01, 1.58)	0.675
NLPR (%), median (IQR)	0.01 (0.01, 0.01)	0.01 (0.01, 0.01)	0.01 (0.01, 0.01)	0.782
SIRI (%), median (IQR)	0.69 (0.49, 1.01)	0.69 (0.48, 1.01)	0.70 (0.49, 1.00)	0.788
AISI (%), median (IQR)	163.50 (108.03, 240.75)	150.35 (105.01, 250.14)	165.50 (110.25, 237.16)	0.477
SII (%), median (IQR)	396.04 (296.66, 533.27)	383.11 (276.55, 546.01)	404.92 (300.25, 528.57)	0.418
Blood sugar(mmol/L), median (IQR)	5.20 (4.75, 5.82)	5.15 (4.75, 5.73)	5.21 (4.75, 5.86)	0.645
Complement C1q(mg/L), median (IQR)	173.10 (150.62, 191.62)	173.40 (150.48, 193.00)	173.05 (150.80, 191.28)	0.999
LDH (U/L), median (IQR)	193.50 (173.00, 217.75)	195.44 (178.00, 219.00)	192.00 (172.00, 216.75)	0.237
SA (mg/dL), median (IQR)	54.03 (49.82, 59.00)	54.03 (49.48, 58.40)	54.03 (50.00, 59.18)	0.583
5'-NT (U/L), median (IQR)	4.00 (3.00, 5.00)	4.00 (3.00, 5.00)	4.00 (3.00, 5.00)	0.606
Pro-GRP (pg/mL), median (IQR)	41.96 (34.69, 45.88)	41.96 (33.72, 44.41)	41.96 (34.82, 46.14)	0.603
SCC (ng/mL), median (IQR)	1.10 (0.78, 1.97)	1.10 (0.73, 1.97)	1.10 (0.78, 1.97)	0.766
Cyfra21-1 (ng/mL), median (IQR)	2.32 (1.79, 2.62)	2.32 (1.87, 2.70)	2.32 (1.78, 2.58)	0.536
CEA (ng/mL), median (IQR)	2.32 (1.74, 2.97)	2.32 (1.89, 3.15)	2.32 (1.68, 2.92)	0.159
CA125 (U/mL), median (IQR)	10.72 (7.62, 11.20)	10.72 (7.76, 12.03)	10.50 (7.54, 10.90)	0.207
NSE (ng/mL), median (IQR)	19.45 (15.72, 20.60)	19.45 (16.30, 20.60)	19.45 (15.60, 20.58)	0.544
Age (years), median (IQR)	61.00 (54.00, 67.00)	61.00 (54.00, 67.00)	61.00 (54.00, 66.75)	0.566
BMI (kg/m2), median (IQR)	25.14 (23.05, 27.18)	25.17 (22.86, 27.19)	25.10 (23.15, 27.17)	0.591
FEV1% predicted (%), median (IQR)	104.36 (93.22, 116.15)	102.90 (88.81, 113.14)	105.29 (94.06, 117.16)	0.063
MVV% predicted (%), median (IQR)	104.06 (88.28, 115.19)	101.32 (85.89, 114.83)	104.89 (90.43, 116.36)	0.038
Maximum diameter (cm), median (IQR)	1.50 (1.20, 1.80)	1.50 (1.20, 1.70)	1.50 (1.20, 1.80)	0.264
CTR (%), median (IQR)	0.50 (0.00, 0.88)	0.56 (0.09, 0.87)	0.46 (0.00, 0.89)	0.194
Ki-67 positive rate (%), median (IQR)	0.00 (0.00, 1.00)	0.00 (0.00, 0.00)	0.00 (0.00, 2.00)	0.283

*COPD* chronic obstructive pulmonary diseases, *ASA* American Society of Anesthesiologists, *PNI* prognostic nutritional index, *NLR* neutrophil–lymphocyte ratio, *PLR* platelet-lymphocyte ratio, *MLR* monocyte-lymphocyte ratio, *dNLR* derived neutrophil-to-lymphocyte ratio, *NLPR* neutrophil to lymphocyte and platelet ratio, *SIRI* systemic inflammatory response syndrome, AISI aggregate index of systemic inflammation, *SII* systemic inflammation index, *LDH* lactate dehydrogenase, *SA* serum amyloid, *5'-NT* 5'-nucleotidase, *Pro-GRP* pro-gastrin-releasing peptide, *SCC* squamous cell carcinoma, *Cyfra21-1* cytokeratin 19-fragments, *CEA* carcinoembryonic antigen, *CA125* carcinoma antigen 125, *NSE* neuron-specific enolase, *BMI* body mass index, *FEV1* forced expiratory volume in one second, *MVV* maximal voluntary ventilation, *CTR* consolidation-to-tumor ratio, *TTF* thyroid transcription factor 1, *PAS* Periodic Acid-Schiff reaction, *PAS-D* Periodic Acid-Schiff reaction with diastase, *CK 5/6* Cytokeratin 5/6, *CK 7* Cytokeratin 7, *MUC-AC* mucin-AC

**Table 2:** Clinical characteristics of patients in the training and validation cohorts

Characteristics	Training Cohort (*n* = 366)			Validation cohort (*n* = 156)		
	**LNM (-) (*n* = 327)**	**LNM ( +) (*n* = 39)**	***p***	**LNM (-) (*n* = 134)**	**LNM ( +) (*n* = 22)**	***p***
Gender, n (%)			0.23			
Female	168 (51.4)	24 (61.5)		78 (58.2)	14 (63.6)	
Male	159 (48.6)	15 (38.5)		56 (41.8)	8 (36.4)	
Hypertension, n (%)			0.297			0.965
No	216 (66.1)	29 (74.4)		92 (68.7)	15 (68.2)	
Yes	111 (33.9)	10 (25.6)		42 (31.3)	7 (31.8)	
Diabetes, n (%)			0.379			0.303
No	286 (87.5)	36 (92.3)		115 (85.8)	17 (77.3)	
Yes	41 (12.5)	3 (7.7)		19 (14.2)	5 (22.7)	
COPD, n (%)			0.548			0.479
No	324 (99.1)	39 (100.0)		131 (97.8)	22 (100.0)	
Yes	3 (0.9)	0 (0.0)		3 (2.2)	0 (0.0)	
Smoking history, n (%)			0.82			0.736
Non-smoker	229 (70.0)	28 (71.8)		99 (73.9)	17 (77.3)	
Smoker	98 (30.0)	11 (28.2)		35 (26.1)	5 (22.7)	
Blood type, n (%)			0.791			0.407
A	94 (28.7)	12 (30.8)		39 (29.1)	5 (22.7)	
B	127 (38.8)	12 (30.8)		46 (34.3)	6 (27.3)	
AB	34 (10.4)	5 (12.8)		14 (10.4)	5 (22.7)	
O	72 (22.0)	10 (25.6)		35 (26.1)	6 (27.3)	
ASA, n (%)			0.331			0.475
1	28 (8.6)	2 (5.1)		9 (6.7)	2 (9.1)	
2	288 (88.1)	34 (87.2)		120 (89.6)	18 (81.8)	
3	11 (3.4)	3 (7.7)		5 (3.7)	2 (9.1)	
Location, n (%)			0.72			0.769
Centrality	40 (12.2)	4 (10.3)		15 (11.2)	2 (9.1)	
Peripherality	287 (87.8)	35 (89.7)		119 (88.8)	20 (90.9)	
Shape, n (%)			0.519			0.907
Regularity	109 (33.3)	11 (28.2)		47 (35.1)	8 (36.4)	
Irregularity	218 (66.7)	28 (71.8)		87 (64.9)	14 (63.6)	
Spiculation, n (%)			0.121			0.069
No	107 (32.7)	8 (20.5)		44 (32.8)	3 (13.6)	
Yes	220 (67.3)	31 (79.5)		90 (67.2)	19 (86.4)	
Cavitation sign, n (%)			0.925			0.132
No	262 (80.1)	31 (79.5)		105 (78.4)	14 (63.6)	
Yes	65 (19.9)	8 (20.5)		29 (21.6)	8 (36.4)	
Calcification, n (%)			0.437			NA
No	322 (98.5)	39 (100.0)		134 (100.0)	22 (100.0)	
Yes	5 (1.5)	0 (0.0)		0 (0.0)	0 (0.0)	
Vascular penetration sign, n (%)			0.37			0.974
No	89 (27.2)	8 (20.5)		37 (27.6)	6 (27.3)	
Yes	238 (72.8)	31 (79.5)		97 (72.4)	16 (72.7)	
Pleural adhesions, n (%)			0.01			0.022
No	128 (39.1)	7 (17.9)		52 (38.8)	3 (13.6)	
Yes	199 (60.9)	32 (82.1)		82 (61.2)	19 (86.4)	
Bronchus sign, n (%)			0.185			0.249
No	227 (69.4)	23 (59.0)		90 (67.2)	12 (54.5)	
Yes	100 (30.6)	16 (41.0)		44 (32.8)	10 (45.5)	
Lobulation, n (%)			0.02			0.065
No	165 (50.5)	12 (30.8)		77 (57.5)	8 (36.4)	
Yes	162 (49.5)	27 (69.2)		57 (42.5)	14 (63.6)	
Lymph node enlargement sign, n (%)			0.251			0.907
No	268 (82.0)	29 (74.4)		111 (82.8)	18 (81.8)	
Yes	59 (18.0)	10 (25.6)		23 (17.2)	4 (18.2)	
Pleural effusion sign, n (%)			0.487			0.564
No	323 (98.8)	39 (100.0)		132 (98.5)	22 (100.0)	
Yes	4 (1.2)	0 (0.0)		2 (1.5)	0 (0.0)	
Lepidic, n (%)			0.049			0.153
No	100 (30.6)	18 (46.2)		35 (26.1)	9 (40.9)	
Yes	227 (69.4)	21 (53.8)		99 (73.9)	13 (59.1)	
Acinar, n (%)			0.638			0.42
No	65 (19.9)	9 (23.1)		21 (15.7)	2 (9.1)	
Yes	262 (80.1)	30 (76.9)		113 (84.3)	20 (90.9)	
Papillary, n (%)			0.319			0.029
No	203 (62.1)	21 (53.8)		82 (61.2)	8 (36.4)	
Yes	124 (37.9)	18 (46.2)		52 (38.8)	14 (63.6)	
Micropapillary, n (%)			0.003			< 0.001
No	273 (83.5)	25 (64.1)		112 (83.6)	11 (50.0)	
Yes	54 (16.5)	14 (35.9)		22 (16.4)	11 (50.0)	
Solid, n (%)			< 0.001			0.015
No	313 (95.7)	31 (79.5)		128 (95.5)	18 (81.8)	
Yes	14 (4.3)	8 (20.5)		6 (4.5)	4 (18.2)	
Mucinous, n (%)			0.015			0.979
No	283 (86.5)	28 (71.8)		116 (86.6)	19 (86.4)	
Yes	44 (13.5)	11 (28.2)		18 (13.4)	3 (13.6)	
CK5/6, n (%)			< 0.001			< 0.001
No	318 (97.2)	26 (66.7)		133 (99.3)	19 (86.4)	
Yes	9 (2.8)	13 (33.3)		1 (0.7)	3 (13.6)	
CK7, n (%)			0.033			0.003
No	252 (77.1)	24 (61.5)		106 (79.1)	11 (50.0)	
Yes	75 (22.9)	15 (38.5)		28 (20.9)	11 (50.0)	
TTF-1, n (%)			0.001			< 0.001
No	241 (73.7)	19 (48.7)		104 (77.6)	9 (40.9)	
Yes	86 (26.3)	20 (51.3)		30 (22.4)	13 (59.1)	
Napsin A, n (%)			< 0.001			< 0.001
No	299 (91.4)	23 (59.0)		119 (88.8)	11 (50.0)	
Yes	28 (8.6)	16 (41.0)		15 (11.2)	11 (50.0)	
MUC-AC, n (%)			0.286			0.526
No	304 (93.0)	38 (97.4)		131 (97.8)	21 (95.5)	
Yes	23 (7.0)	1 (2.6)		3 (2.2)	1 (4.5)	
P63, n (%)			< 0.001			< 0.001
No	310 (94.8)	25 (64.1)		131 (97.8)	17 (77.3)	
Yes	17 (5.2)	14 (35.9)		3 (2.2)	5 (22.7)	
CyclinD1, n (%)			0.057			< 0.001
No	315 (96.3)	35 (89.7)		129 (96.3)	14 (63.6)	
Yes	12 (3.7)	4 (10.3)		5 (3.7)	8 (36.4)	
EMA, n (%)			< 0.001			< 0.001
No	319 (97.6)	32 (82.1)		130 (97.0)	15 (68.2)	
Yes	8 (2.4)	7 (17.9)		4 (3.0)	7 (31.8)	
CD31, n (%)			0.023			< 0.001
No	313 (95.7)	34 (87.2)		129 (96.3)	15 (68.2)	
Yes	14 (4.3)	5 (12.8)		5 (3.7)	7 (31.8)	
D2-40, n (%)			< 0.001			< 0.001
No	315 (96.3)	32 (82.1)		129 (96.3)	16 (72.7)	
Yes	12 (3.7)	7 (17.9)		5 (3.7)	6 (27.3)	
Stretch fiber, n (%)			0.008			0.043
No	242 (74.0)	21 (53.8)		101 (75.4)	12 (54.5)	
Yes	85 (26.0)	18 (46.2)		33 (24.6)	10 (45.5)	
PAS, n (%)			< 0.001			0.005
No	305 (93.3)	21 (53.8)		124 (92.5)	16 (72.7)	
Yes	22 (6.7)	18 (46.2)		10 (7.5)	6 (27.3)	
PAS-D, n (%)			0.001			< 0.001
No	303 (92.7)	30 (76.9)		127 (94.8)	14 (63.6)	
Yes	24 (7.3)	9 (23.1)		7 (5.2)	8 (36.4)	
Albumin (g/L), median (IQR)	60.40 (58.30, 62.50)	58.80 (56.30, 60.40)	0.004	59.60 (57.70, 61.90)	58.55 (57.62, 60.13)	0.191
Lymphocyte (× 109/L), median (IQR)	1.81 (1.46, 2.21)	1.59 (1.28, 1.85)	0.015	1.83 (1.42, 2.27)	1.63 (1.43, 2.01)	0.326
PNI (%), median (IQR)	69.65 (66.45, 72.25)	66.00 (64.55, 69.22)	< 0.001	69.05 (66.01, 71.39)	67.28 (64.33, 69.30)	0.072
Neutrophil (× 109/L), median (IQR)	2.94 (2.45, 3.90)	3.02 (2.64, 3.39)	0.934	3.07 (2.44, 3.85)	3.05 (2.81, 3.95)	0.341
Eosinophil (× 109/L), median (IQR)	0.11 (0.06, 0.18)	0.11 (0.07, 0.21)	0.689	0.11 (0.07, 0.21)	0.10 (0.07, 0.15)	0.402
Basophil (× 109/L), median (IQR)	0.03 (0.02, 0.04)	0.03 (0.02, 0.04)	0.716	0.03 (0.02, 0.04)	0.03 (0.03, 0.04)	0.524
Monocyte (× 109/L), median (IQR)	0.42 (0.34, 0.51)	0.40 (0.33, 0.52)	0.994	0.42 (0.33, 0.50)	0.42 (0.36, 0.50)	0.704
Erythrocyte (× 1012/L), median (IQR)	4.51 (4.24, 4.82)	4.50 (4.20, 4.82)	0.898	4.48 (4.09, 4.85)	4.58 (4.34, 4.86)	0.367
Hemoglobin (g/L), median (IQR)	138.00 (129.00, 149.00)	139.00 (125.00, 148.00)	0.409	136.50 (126.00, 145.75)	137.50 (126.50, 145.00)	0.923
Platelet (× 109/L), median (IQR)	232.00 (198.50, 264.50)	261.00 (231.50, 288.50)	0.013	230.00 (191.00, 264.00)	242.50 (211.25, 286.00)	0.179
NLR (%), median (IQR)	1.70 (1.26, 2.23)	1.91 (1.57, 2.24)	0.042	1.66 (1.27, 2.12)	2.03 (1.70, 2.43)	0.026
PLR (%), median (IQR)	130.66 (104.47, 159.12)	161.74 (136.77, 181.94)	< 0.001	127.15 (93.63, 157.73)	155.21 (116.53, 180.74)	0.043
MLR (%), median (IQR)	0.23 (0.18, 0.29)	0.27 (0.22, 0.32)	0.012	0.22 (0.18, 0.28)	0.25 (0.20, 0.33)	0.182
dNLR (%), median (IQR)	1.28 (1.01, 1.58)	1.40 (1.12, 1.60)	0.147	1.24 (0.97, 1.57)	1.51 (1.28, 1.69)	0.012
NLPR (%), median (IQR)	0.01 (0.01, 0.01)	0.01 (0.01, 0.01)	0.496	0.01 (0.01, 0.01)	0.01 (0.01, 0.01)	0.212
SIRI (%), median (IQR)	0.69 (0.47, 1.02)	0.80 (0.63, 0.92)	0.068	0.65 (0.47, 0.98)	0.79 (0.57, 1.33)	0.108
AISI (%), median (IQR)	163.03 (105.84, 232.89)	221.50 (144.41, 264.92)	0.017	147.89 (100.00, 242.84)	181.92 (135.84, 375.84)	0.056
SII (%), median (IQR)	389.76 (298.64, 511.21)	519.12 (343.84, 622.08)	0.006	369.06 (273.13, 515.99)	504.51 (390.20, 594.27)	0.013
Blood sugar(mmol/L), median (IQR)	5.22 (4.78, 5.86)	5.10 (4.62, 5.76)	0.172	5.11 (4.73, 5.67)	5.48 (4.96, 6.27)	0.117
Complement C1q(mg/L), median (IQR)	171.00 (149.90, 188.55)	189.20 (163.10, 206.95)	0.002	170.80 (149.72, 192.62)	179.80 (168.00, 192.75)	0.089
LDH (U/L), median (IQR)	191.00 (172.00, 215.50)	195.89 (173.00, 229.50)	0.209	193.00 (176.50, 219.75)	196.50 (184.75, 207.00)	0.996
SA (mg/dL), median (IQR)	54.00 (49.75, 58.80)	57.10 (51.30, 63.95)	0.033	54.03 (49.12, 58.58)	56.00 (52.37, 58.25)	0.151
5'-NT (U/L), median (IQR)	4.00 (3.00, 5.00)	4.00 (3.00, 4.62)	0.497	4.00 (3.00, 5.00)	4.00 (4.00, 5.00)	0.149
Pro-GRP (pg/mL), median (IQR)	41.96 (34.69, 45.59)	41.96 (35.90, 49.95)	0.335	41.96 (32.44, 44.34)	41.96 (41.34, 45.13)	0.069
SCC (ng/mL), median (IQR)	1.10 (0.78, 1.97)	1.10 (0.94, 1.96)	0.706	1.06 (0.72, 1.97)	1.36 (0.76, 1.97)	0.273
Cyfra21-1 (ng/mL), median (IQR)	2.32 (1.80, 2.58)	2.32 (1.73, 2.57)	0.697	2.32 (1.88, 2.70)	2.32 (1.72, 2.32)	0.674
CEA (ng/mL), median (IQR)	2.32 (1.75, 2.92)	2.32 (1.09, 2.78)	0.251	2.32 (1.80, 2.97)	2.32 (2.32, 3.83)	0.109
CA125 (U/mL), median (IQR)	10.30 (7.40, 10.72)	10.72 (9.84, 12.80)	0.002	10.71 (7.61, 11.28)	11.41 (10.72, 14.02)	0.002
NSE (ng/mL), median (IQR)	19.45 (15.50, 20.05)	19.45 (16.95, 22.95)	0.239	19.45 (15.93, 20.60)	19.45 (18.27, 20.16)	0.488
Age (years), median (IQR)	62.00 (54.00, 67.00)	56.00 (48.50, 64.00)	0.017	62.00 (54.25, 68.00)	56.00 (50.75, 63.50)	0.034
BMI (kg/m2), median (IQR)	24.97 (23.04, 27.04)	26.37 (24.53, 29.94)	0.005	24.91 (22.59, 27.02)	25.41 (24.25, 28.07)	0.069
FEV1% predicted (%), median (IQR)	105.30 (94.90, 117.40)	97.01 (85.34, 109.50)	0.037	102.69 (88.96, 114.94)	104.15 (85.17, 110.00)	0.563
MVV% predicted (%), median (IQR)	105.23 (90.91, 117.04)	99.47 (87.00, 111.57)	0.126	101.32 (85.28, 114.90)	99.50 (86.96, 114.40)	0.776
Maximum diameter (cm), median (IQR)	1.50 (1.20, 1.75)	1.60 (1.50, 1.90)	0.001	1.45 (1.10, 1.60)	1.60 (1.40, 1.95)	0.013
CTR (%), median (IQR)	0.43 (0.00, 0.85)	0.85 (0.40, 1.00)	< 0.001	0.50 (0.00, 0.73)	0.79 (0.60, 1.00)	0.004
Ki-67 positive rate (%), median (IQR)	0.00 (0.00, 1.25)	0.00 (0.00, 15.00)	0.103	0.00 (0.00, 0.00)	0.00 (0.00, 14.38)	0.002

*LNM(* +*)* positive for lymph node metastasis, *LNM(-)* negative for lymph node metastasis, *COPD* chronic obstructive pulmonary diseases, *ASA* American Society of Anesthesiologists, *PNI* prognostic nutritional index, *NLR* neutrophil–lymphocyte ratio, *PLR* platelet-lymphocyte ratio, *MLR* monocyte-lymphocyte ratio, *Dnlr* derived neutrophil-to-lymphocyte ratio, *NLPR* neutrophil to lymphocyte and platelet ratio, *SIRI* systemic inflammatory response syndrome, *AISI* aggregate index of systemic inflammation, *SII* systemic inflammation index, *LDH* lactate dehydrogenase, *SA* serum amyloid, *5'-NT* 5'-nucleotidase, *Pro-GRP* pro-gastrin-releasing peptide, *SCC* squamous cell carcinoma, *Cyfra21-1* cytokeratin 19-fragments, *CEA* carcinoembryonic antigen, *CA125* carcinoma antigen 125, *NSE* neuron-specific enolase, *BMI* body mass index, *FEV1* forced expiratory volume in one second, *MVV* maximal voluntary ventilation, *CTR* consolidation-to-tumor ratio, *TTF* thyroid transcription factor 1, *PAS* Periodic Acid-Schiff reaction, *PAS-D* Periodic Acid-Schiff reaction with diastase, *CK 5/6* Cytokeratin 5/6, *CK 7* Cytokeratin 7, *MUC-AC* mucin-AC

### Identifying risk factors for lymph node metastasis

Univariate and then multivariate logistic regression analyses were performed in the training cohort to investigate independent risk factors for lymph node metastasis, and the results of the logistic regression analyses are shown in Table 3.

**Table 3:** Univariate and multivariate logistic regression analysis of LNM factors in a training cohort

Characteristics	Univariate analysis		Multivariate analysis	
	**OR (95%CI)**	***P***	**OR (95%CI)**	***P***
Age	0.959 (0.927, 0.991)	0.013	0.934 (0.871, 0.996)	< 0.001
SA	1.041 (1.003, 1.081)	0.033	1.025 (0.937, 1.109)	0.008
CA125	1.045 (1.006, 1.094)	0.028	1.103 (1.021, 1.189)	0.042
Mucinous				
No	Ref	Ref	Ref	Ref
Yes	2.527 (1.135, 5.324)	0.018	1.729 (0.371, 7.519)	0.003
Napsin A				
No	Ref	Ref	Ref	Ref
Yes	7.429 (3.494, 15.681)	< 0.001	2.704 (0.489, 15.541)	0.007
CK5/6				
No	Ref	Ref	Ref	Ref
Yes	17.667 (6.993, 46.668)	< 0.001	18.668 (2.938, 154.991)	0.042
PAS-D				
No	Ref	Ref	Ref	Ref
Yes	3.788 (1.548, 8.676)	0.002	3.521 (0.605, 19.102)	0.067
CK7				
No	Ref	Ref	Ref	Ref
Yes	2.100 (1.030, 4.171)	0.036	0.146 (0.021, 0.935)	0.123
PAS				
No	Ref	Ref	Ref	Ref
Yes	11.883 (5.542, 25.739)	< 0.001	1.673 (0.307, 7.799)	0.148
Pleural adhesions				
No	Ref	Ref	Ref	Ref
Yes	2.940 (1.332, 7.436)	0.013	3.516 (0.800, 19.883)	0.21
D2-40				
No	Ref	Ref	Ref	Ref
Yes	5.742 (2.015, 15.355)	0.001	4.325 (0.285, 89.145)	0.252
TTF-1				
No	Ref	Ref	Ref	Ref
Yes	2.950 (1.500, 5.827)	0.002	3.817 (0.668, 21.137)	0.253
CD31				
No	Ref	Ref	Ref	Ref
Yes	3.288 (1.013, 9.196)	0.031	0.393 (0.013, 10.772)	0.3
Solid				
No	Ref	Ref	Ref	Ref
Yes	5.770 (2.159, 14.602)	< 0.001	1.925 (0.246, 13.577)	0.47
Micropapillary				
No	Ref	Ref	Ref	Ref
Yes	2.831 (1.355, 5.735)	0.004	2.189 (0.495, 9.065)	0.515
Stretch fiber				
No	Ref	Ref	Ref	Ref
Yes	2.440 (1.231, 4.801)	0.01	2.134 (0.578, 7.992)	0.528
EMA, n (%)				
No	Ref	Ref	Ref	Ref
Yes	8.723 (2.889, 25.881)	< 0.001	2.237 (0.213, 22.148)	0.588
P63				
No	Ref	Ref	Ref	Ref
Yes	10.212 (4.487, 23.219)	< 0.001	9.324 (1.994, 52.880)	0.608
Lobulation				
No	Ref	Ref	Ref	Ref
Yes	2.292 (1.146, 4.838)	0.023	2.316 (0.647, 9.247)	0.989
Maximum Diameter	5.159 (1.965, 14.589)	0.001	3.651 (0.759, 20.700)	0.119
FEV1% predicted	0.982 (0.964, 1.000)	0.045	0.979 (0.946, 1.013)	0.12
PLR	1.010 (1.004, 1.016)	0.001	0.994 (0.954, 1.029)	0.067
Lymphocyte	0.475 (0.235, 0.896)	0.03	0.087 (0.001, 2.061)	0.199
BMI	1.172 (1.067, 1.288)	0.001	1.367 (1.161, 1.642)	0.21
CTR	5.724 (2.351, 15.258)	< 0.001	0.988 (0.171, 5.685)	0.284
PNI	0.925 (0.876, 0.977)	0.005	NA (NA, NA)	0.352
Ki-67 positive rate	1.029 (1.010, 1.046)	0.001	0.990 (0.949, 1.028)	0.495
Complement C1q	1.018 (1.007, 1.029)	0.001	1.020 (0.999, 1.042)	0.553
Platelet	1.006 (1.001, 1.011)	0.016	1.012 (0.989, 1.041)	0.747
Albumin	0.942 (0.888, 1.003)	0.047	1.010 (0.896, 1.176)	0.886
Acinar				
No	Ref	Ref		
Yes	0.827 (0.388, 1.926)	0.639		
ASA				
1	Ref	Ref		
2	1.653 (0.467, 10.515)	0.505		
3	3.818 (0.561, 32.139)	0.171		
Blood type				
A	Ref	Ref		
B	0.740 (0.315, 1.736)	0.484		
AB	1.152 (0.346, 3.360)	0.804		
O	1.088 (0.436, 2.662)	0.853		
Bronchus sign				
No	Ref	Ref		
Yes	1.579 (0.788, 3.099)	0.188		
Calcification				
No	Ref	Ref		
Yes	0.000 (NA, NA)	0.989		
Cavitation sign				
No	Ref	Ref		
Yes	1.040 (0.429, 2.270)	0.925		
COPD				
No	Ref	Ref		
Yes	0.000 (NA, NA)	0.987		
CyclinD1				
No	Ref	Ref		
Yes	3.000 (0.805, 9.151)	0.069		
Diabetes				
No	Ref	Ref		
Yes	0.581 (0.136, 1.708)	0.384		
Gender				
Female	Ref	Ref		
Male	0.660 (0.328, 1.292)	0.232		
Hypertension				
No	Ref	Ref		
Yes	0.671 (0.301, 1.384)	0.3		
Lepidic				
No	Ref	Ref		
Yes	0.514 (0.262, 1.015)	0.052		
Location				
Centrality	Ref	Ref		
Peripherality	1.220 (0.456, 4.238)	0.72		
Lymph node enlargement sign				
No	Ref	Ref		
Yes	1.566 (0.693, 3.295)	0.255		
MUC-AC				
No	Ref	Ref		
Yes	0.348 (0.019, 1.726)	0.308		
Papillary				
No	Ref	Ref		
Yes	1.403 (0.713, 2.737)	0.32		
Pleural effusion sign				
No	Ref	Ref		
Yes	0.000 (NA, NA)	0.985		
Shape				
Regularity	Ref	Ref		
Irregularity	1.273 (0.626, 2.758)	0.52		
Smoking history				
Non-smoker	Ref	Ref		
Smoker	0.918 (0.423, 1.870)	0.82		
Spiculation				
No	Ref	Ref		
Yes	1.885 (0.876, 4.528)	0.126		
Vascular penetration sign				
No	Ref	Ref		
Yes	1.449 (0.671, 3.492)	0.372		
Cyfra21-1	0.934 (0.623, 1.078)	0.684		
AISI	1.000 (0.999, 1.001)	0.495		
Basophil	0.501 (0.000, 48.590)	0.856		
Blood Sugar	0.802 (0.556, 1.072)	0.187		
CEA	0.939 (0.717, 1.151)	0.602		
dNLR	1.062 (0.636, 1.543)	0.778		
Eosinophil	1.375 (0.221, 4.520)	0.618		
Erythrocyte	0.920 (0.431, 1.956)	0.829		
Hemoglobin	0.981 (0.959, 1.002)	0.078		
LDH	1.006 (0.998, 1.015)	0.122		
MLR	1.803 (0.830, 3.913)	0.096		
Monocyte	1.168 (0.731, 1.673)	0.366		
MVV% predicted	0.987 (0.971, 1.001)	0.097		
Neutrophil	0.945 (0.704, 1.185)	0.668		
NLPR	0.000 (NA, NA)	0.597		
NLR	1.023 (0.775, 1.215)	0.825		
NSE	1.029 (0.988, 1.067)	0.133		
Pro_GRP	1.005 (0.979, 1.028)	0.695		
SCC	0.960 (0.562, 1.451)	0.867		
SII	1.000 (1.000, 1.001)	0.265		
SIRI	1.023 (0.799, 1.170)	0.781		
5'-NT	0.995 (0.747, 1.271)	0.973		

*LNM* lymph node metastasis, *COPD* chronic obstructive pulmonary diseases, *ASA* American Society of Anesthesiologists, *PNI* prognostic nutritional index, *NLR* neutrophil–lymphocyte ratio, *PLR* platelet-lymphocyte ratio, *MLR* monocyte-lymphocyte ratio, *dNLR* derived neutrophil-to-lymphocyte ratio, *NLPR* neutrophil to lymphocyte and platelet ratio, *SIRI* systemic inflammatory response syndrome, *AISI* aggregate index of systemic inflammation, *SII* systemic inflammation index, *PIV* pan-immune-inflammation value, *LDH* lactate dehydrogenase, *SA* serum amyloid, *5'-NT* 5'-nucleotidase, *Pro-GRP* pro-gastrin-releasing peptide, *SCC* squamous cell carcinoma, *Cyfra21-1* cytokeratin 19-fragments, *CEA* carcinoembryonic antigen, *CA125* carcinoma antigen 125, *NSE* neuron-specific enolase, *BMI* body mass index, *FEV1* forced expiratory volume in one second, *MVV* maximal voluntary ventilation, *CTR* consolidation-to-tumor ratio, *TTF* thyroid transcription factor 1, *PAS* Periodic Acid-Schiff reaction, *PAS-D* Periodic Acid-Schiff reaction with diastase, *CK 5/6* Cytokeratin 5/6, *CK 7* Cytokeratin 7, *MUC-AC* mucin-AC

**Fig. 2:**
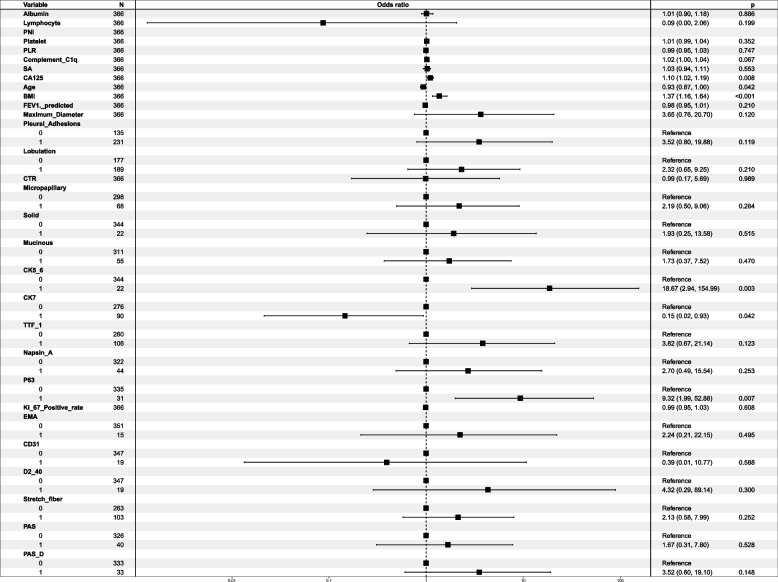
Multi-factor logistic regression analysis of forest plots. PNI, prognostic nutritional index; PLR, platelet-lymphocyte ratio; SA, serum amyloid; CA125, carcinoma antigen 125; BMI, body mass index; FEV1, forced expiratory volume in one second; TTF, thyroid transcription factor 1; PAS, Periodic Acid-Schiff reaction; PAS-D, Periodic Acid-Schiff reaction with diastase; CK 5/6, Cytokeratin 5/6; CK 7, Cytokeratin 7; MUC-AC, mucin-AC

Univariate analysis showed that as many as 30 factors were potential risk factors for lymph node metastasis in early-stage small lung adenocarcinoma (*P* < 0.05). After further multivariate logistic regression analysis, six indicators were finally identified to be independently associated with lymph node metastasis. The six indicators were: age [odds ratio (OR) = 0.934; 95% confidence interval (CI): 0.871–0.996; *P* < 0.001]; SA (OR = 1.025; 95% CI: 0.937–1.109; *P* = 0.008); CA125 (OR = 1.103; 95% CI: 1.021–1.189; *P* = 0.042); Mucinous (no and yes; OR = 1.729; 95% CI: 0.371–7.519; *P* = 0.003); Napsin A (no and yes; OR = 2.704; 95% CI: 0.489–15.541; *P* = 0.007); and CK5/6 (no and yes; OR = 18.668; 95% CI: 2.938–154.991; *P* = 0.042). The results of the multifactorial logistic regression analysis of the 30 factors screened in this study are detailed in the forest plot (Fig. 2).

### Frequency of targeted gene alterations

Of the 522 patients, 46 underwent genetic alteration analysis using ARMS-PCR. Of these, 37 (80.4%) samples had gene mutations detected. The mutation frequencies of EGFR and KRAS genes were 71.7% (33/46) and 8.7% (4/46), respectively. EGFR mutations were the most common type of alteration, with 39.1% (18/46) of patients having mutations in Exon21, 26.1% (12/46) having mutations in Exon19, 2.2% (1/46) having mutations in Exon18, 2.2% (1/46) having mutations in Exon20, and 2.2% (1/46) having double mutations in Exon18 and Exon20. All of the KRAS mutations were mutations in Exon2, with a total of 4 cases or 8.7% (4/46). Of the 37 patients with genetic mutations, 4 had lymph node metastases and 33 did not. Considering the possibility of gene mutations in patients without genetic testing, this study will not include gene mutations in the univariate and multifactorial analyses, but will simply elaborate the findings.

### Nomogram construction

**Fig. 3:**
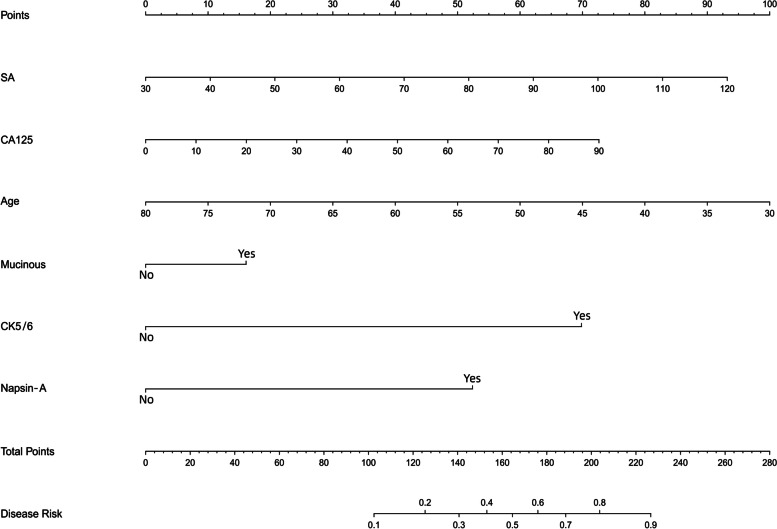
Nomogram for predicting the probability of LNM in small invasive lung adenocarcinoma. SA, serum amyloid; CA125, carcinoma antigen 125; CK 5/6, Cytokeratin 5/6. As shown in this nomogram, there are 9 axes, and axes 2–7 represent the six variables in the prediction model. By drawing a line perpendicular to the highest point axis, the estimated score for each risk factor can be calculated and can be further summed to obtain a total score. The total score axis is then used to predict the probability of developing lymph node metastasis in invasive lung adenocarcinoma, which in turn can further guide the surgical approach

All six independent risk factors for lymph node metastasis in small invasive lung adenocarcinoma within 2 cm were included to create a logistic regression model. The probability of lymph node metastasis in small invasive lung adenocarcinoma could be calculated by the following formula: ln (p/1-p) = -0.068 × age + 0.025 × SA + 0.098 × CA125 + 0.547 × mucinous (no = 0; yes = 1) + 2.927 × CK5/6 (no = 0; yes = 1)—13.972. Based on the above equation, a nomogram of the predicted probability of lymph node metastasis in invasive lung adenocarcinoma within 2 cm was plotted using R statistical software (Fig. 3). As shown in this nomogram, there are 9 axes, and axes 2–7 represent the six variables in the prediction model. By drawing a line perpendicular to the highest point axis, the estimated score for each risk factor can be calculated and can be further summed to obtain a total score. The total score axis is then used to predict the probability of developing lymph node metastasis in invasive lung adenocarcinoma, which in turn can further guide the surgical approach.

### Predictive performance and validation of the nomogram

**Fig. 4:**
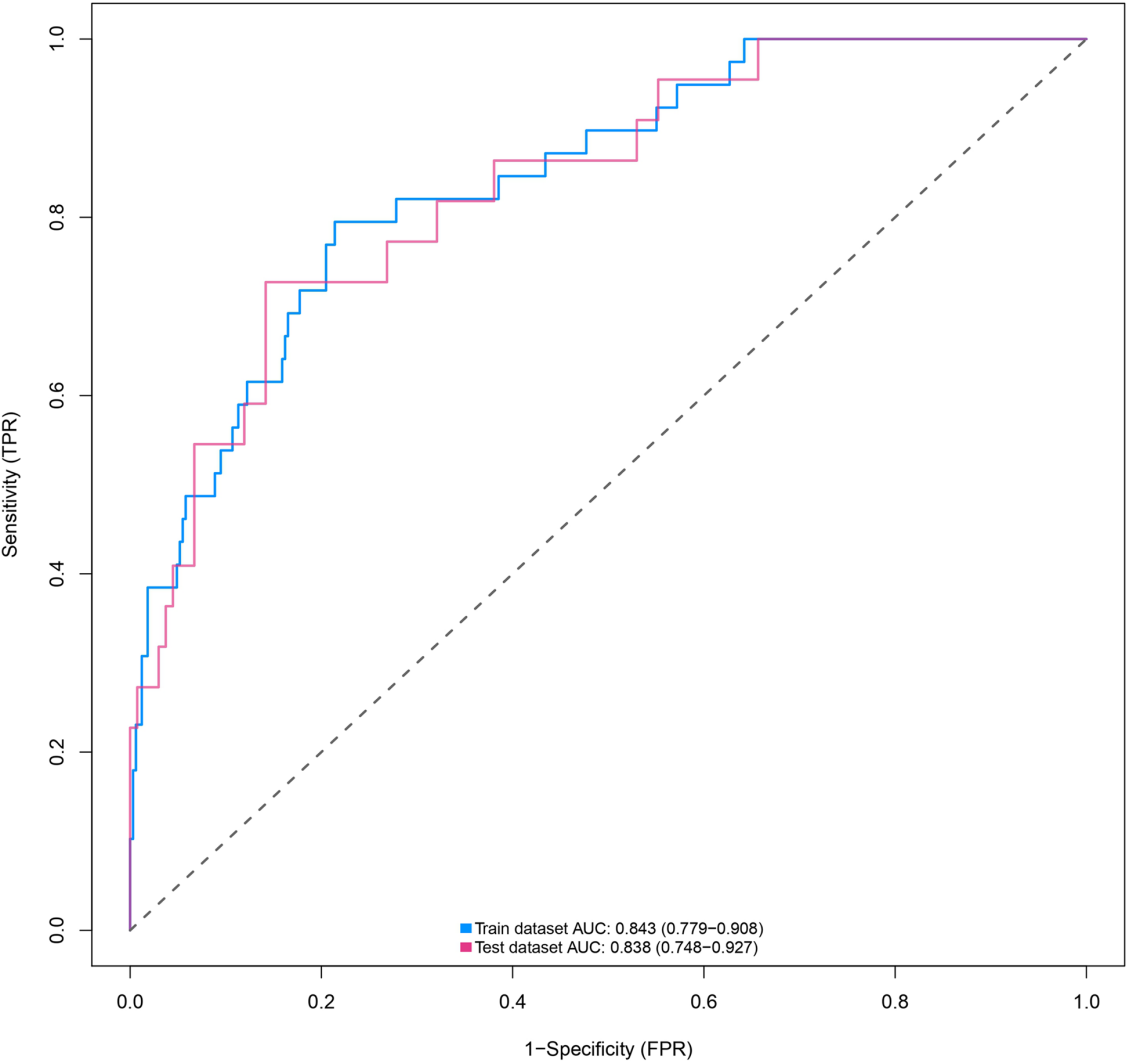
Results of ROC curve in the training and validation cohorts

**Fig. 5:**
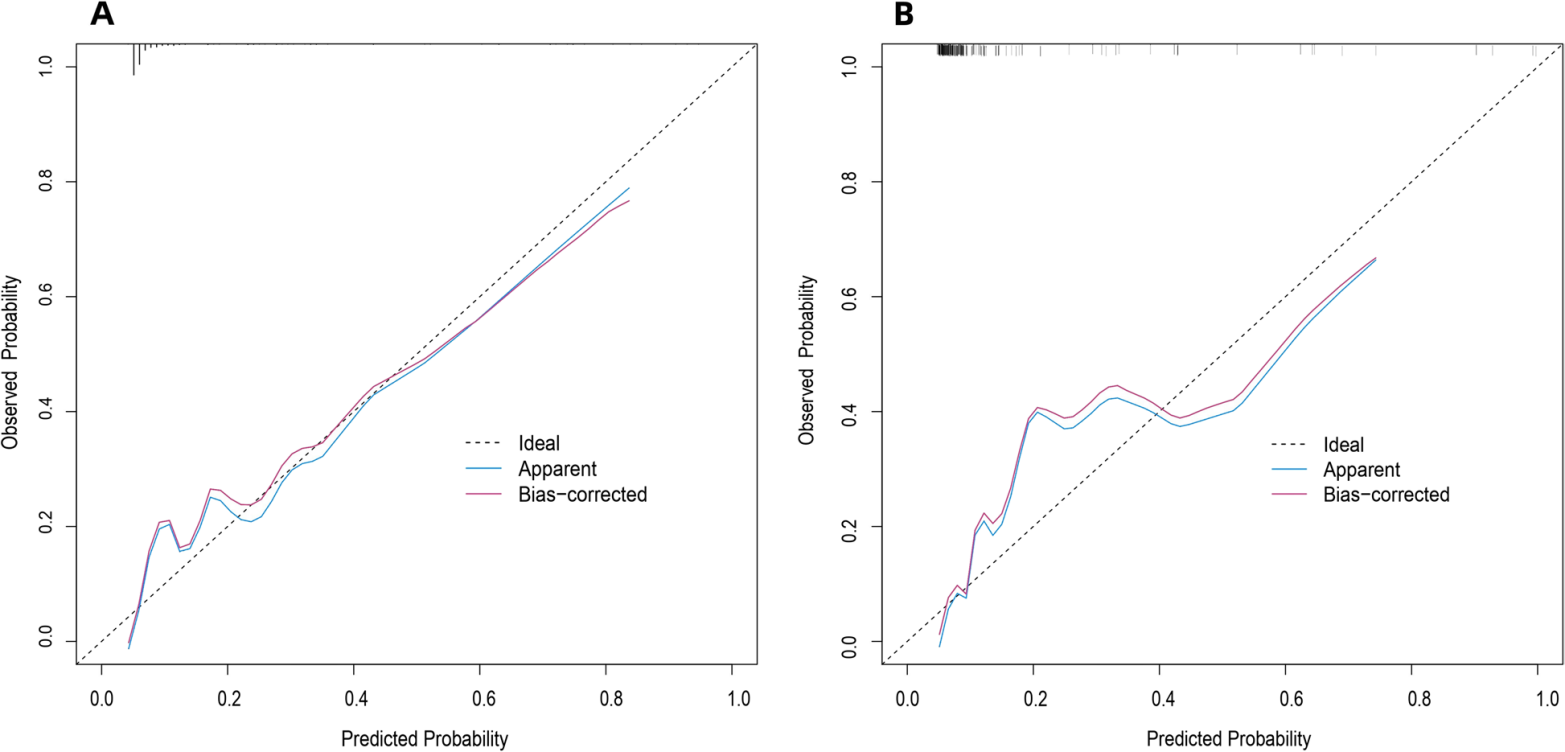
**A, B** Calibration curves of the prediction nomogram in the training cohort (**A**) and validation cohort (**B**). The X-axis represents the probability predicted by the nomogram and the Y-axis represents the actual probability of LNM in invasive lung adenocarcinoma within 2 cm. The black dashed line represents the ideal curve, the blue solid line represents the apparent curve (uncorrected), and the red solid line represents the deviation curve corrected by bootstrap method (B = 1000 times). LNM, lymph node metastasis

Discrimination ability of the prediction model and nomogram is assessed by the ROC curve (Fig. 4). ROC area under the curve (AUC) was 0.843 (95% CI: 0.779–0.908) for the training cohort and 0.838 (95% CI: 0.748–0.927) for the validation cohort, indicating that the nomogram has good predictive accuracy. The ROC curve for the training cohort had a threshold of 0.089 and sensitivities and specificities of 0.795 and 0.786, respectively (Table 4). Our Hosmer–Lemeshow test and calibration charts were used to assess calibration capability. Our *p*-value for the Hosmer–Lemeshow test was 0.0613 in the training cohort and 0.8628 in the validation cohort, indicating that the difference between the predicted and actual observed probabilities was negligible. A good calibration of the prediction nomogram is also demonstrated by the calibration plots of the training cohort (Fig. 5A) and the validation cohort (Fig. 5B). The bias-corrected C-index for the training cohort was 0.8444 and the bias-corrected C-index for the validation cohort was 0.8375, further demonstrating the goodness of the prediction model.

**Table 4:** Results of ROC curve for training cohort

Characteristics	Value
Threshold	0.089
Specificity	0.786
Sensitivity	0.795
Accuracy	0.787
TN	257
TP	31
FN	8
FP	70
NPV	0.97
PPV	0.307
FDR	0.693
FPR	0.214
TPR	0.795
TNR	0.786
FNR	0.205
1-specificity	0.214
1-sensitivity	0.205
1-accuracy	0.213
1-npv	0.03
1-ppv	0.693
Precision	0.307
Recall	0.795
Youden	1.581
Closest.topleft	0.088

*TP* true positive, *FP* false positive, *TN* true negative, *FN* false negative, *TPR* true positive rate, *FPR* false positive rate, *TNR* true negative rate, *FNR* false negative rate, *PPV* positive predict value, *NPR* negative predict value, *FDR* false discovery rate

### Clinical utility of the predictive nomogram

**Fig. 6:**
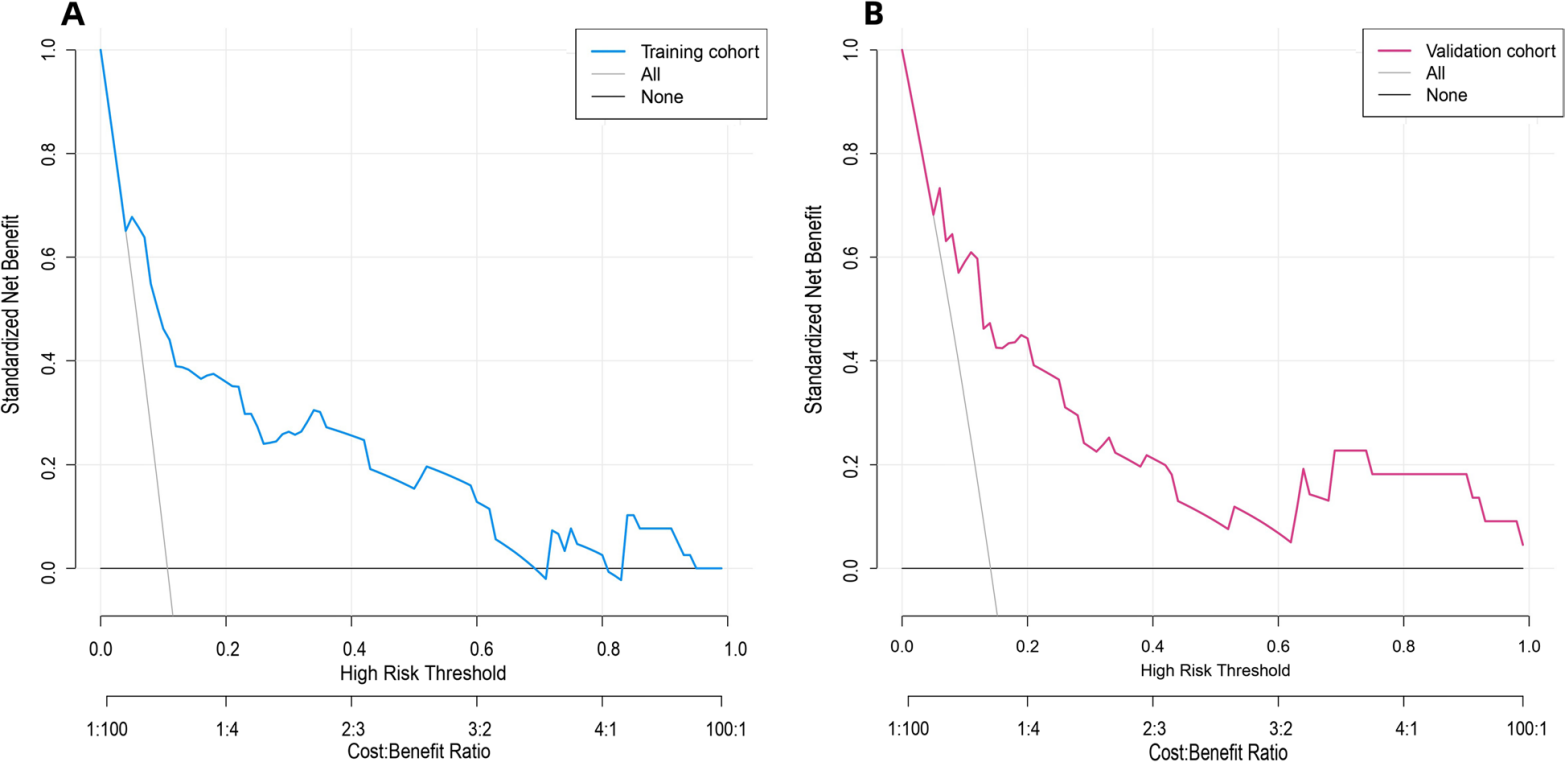
**A, B** Decision curve analysis of predicted nomogram in the training cohort (**A**) and validation cohort (**B**). The y-axis measures the net benefit, the black line represents the hypothesis that no lymph node metastasis has occurred in invasive lung adenocarcinoma within 2 cm, and the gray line represents the hypothesis that lymph node metastasis has occurred in invasive lung adenocarcinoma measuring ≤ 2 cm. The blue line in Fig. 6A represents the training cohort, and the red line in Fig. 6B represents the validation cohort

**Fig. 7:**
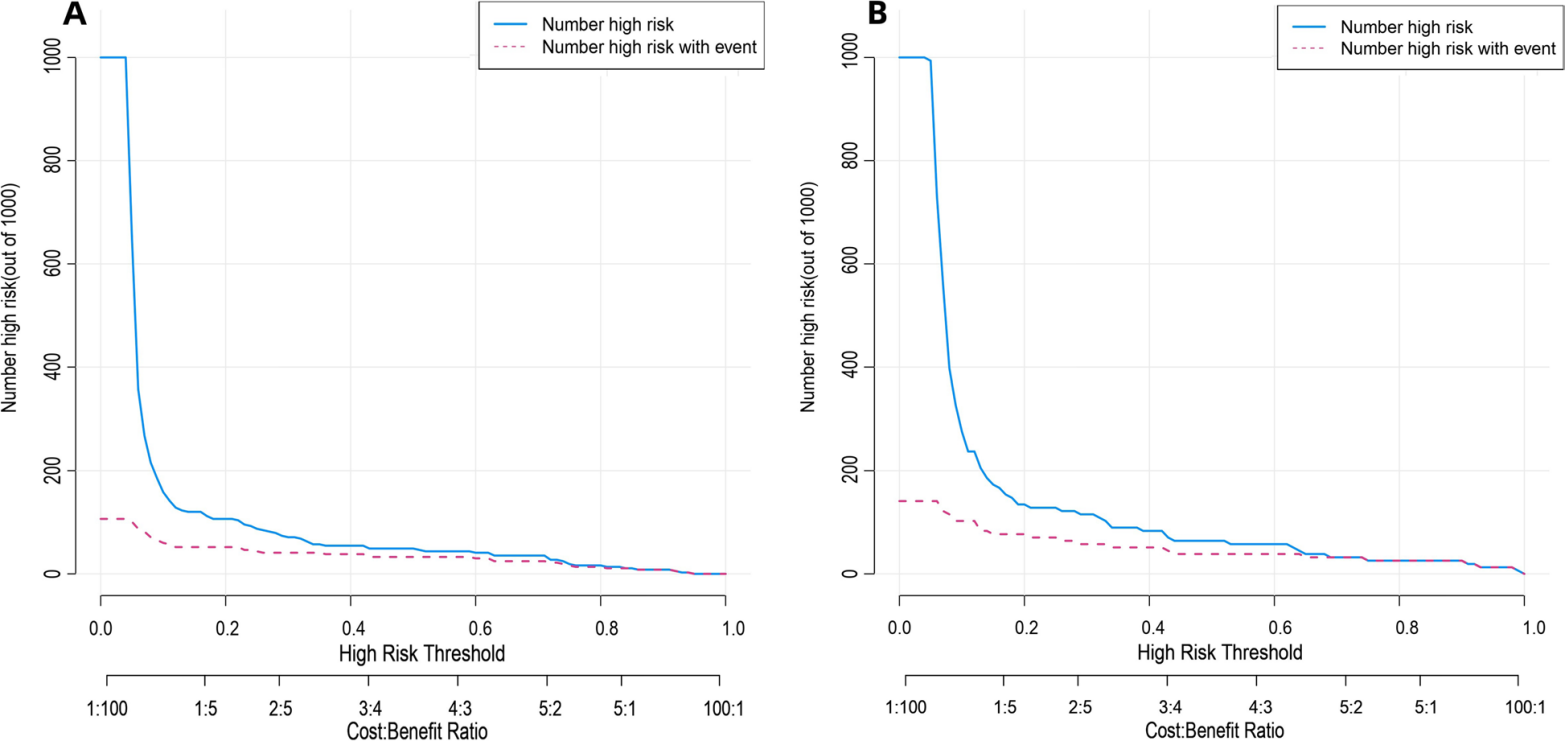
**A, B** Clinical impact curves of predicted nomogram in the training cohort (**A**) and validation cohort (**B**). The horizontal coordinate is the probability threshold and the vertical coordinate is the number of people. The blue line indicates the number of people judged by the model to have lymph node metastasis at different probability thresholds; the red line indicates the number of people judged by the model to be at high risk and to have true lymph node metastasis at different probability thresholds. At the bottom, a cost: benefit ratio is also added, indicating the ratio of loss to benefit at different probability thresholds

Just as shown in Fig. 6A and B, DCA was used to assess the clinical utility of the prediction nomogram. Findings show that the nomogram provided greater net benefit and broader threshold probabilities for predicting the risk of lymph node metastasis in invasive lung adenocarcinoma within 2 cm in both the training and validation cohorts, showing that the nomogram is clinically useful. Figure 7A and B show the clinical impact curves (CIC) for the validation cohort and the verification cohort, respectively. The curves show that a high benefit ratio is obtained within a probability threshold of 0.2–1.0. It suggests that the present model can indeed be used clinically to predict the probability of lymph node metastasis in small invasive lung adenocarcinoma.

## Discussion

In this retrospective study, we developed a nomogram to predict the incidence of lymph node metastasis. In this study, age, SA, CA125, mucin composition, CK5/6, and napsin-A were found to be independent risk factors for lymph node metastasis. The results of genetic testing showed that EGFR was the most common alteration. A nomogram model was developed to assess the risk of lymph node metastasis, which showed consistent discriminatory performance and satisfactory calibration. In 2012, a related study by Terumoto Koike et al. identified the following four predictors of mediastinal lymph node metastasis: (age ≥ 67 years, CEA ≥ 3.5 ng/ml, tumor size ≥ 2.0 cm, and the CTR ≥ 89%) [26]. Advanced age was a common predictor in both our studies. As for hematologic components, our study showed SA and CA125 as predictors. CTR and tumor size were not shown to be associated with mediastinal lymph node metastasis in our study. The inclusion of immunologic components in the predictors is an innovative point of our study. These previously unpublished observations have potential implications for the therapeutic management of early-stage lung adenocarcinoma. This is because the nomogram may have the potential to predict lymph node status before the end of surgery and to guide surgeons in developing lymph node dissection strategies.

Many studies have been conducted on the effect of age on lymph node metastasis in non-small cell lung cancer [26, 38–46]. A part of the findings concluded that youth is an influential factor for lymph node metastasis in lung cancer, with a higher risk of lymph node metastasis in lung cancer patients at a younger age [26, 41–43]. Another part of the study showed that age had no significant effect on lymph node metastasis in lung cancer patients [44–46]. This discrepancy may be due to differences in the patients included in the study, sample size, and analysis methods. Therefore, the different conclusions reached in previous studies are explainable and acceptable. Based on our findings, we conclude that patients with young invasive lung adenocarcinoma are at greater risk for lymph node metastasis and require more thorough and meticulous lymph node dissection.

To date, there have been some case reports of elevated levels of SA being associated with lung cancer [47–49]. The predominance of salivary amylase was observed in these studies from the amylase isozyme pattern in serum and tumor tissues. Amylase levels were higher in tumor tissue than in normal lung tissue. Immunohistochemical studies revealed that amylase was located in tumor cells. Observation of ultrastructure revealed electron-dense particles in the cytoplasm of tumor cells. The findings suggest that in this case, amylase is produced by lung cancer. The possibility that serum amylase levels may be a highly sensitive marker for lung cancer was raised in these studies. Our findings found that lung adenocarcinoma patients with high levels of SA concentration in the blood had a higher risk of lymph node metastasis.

CA125 has long been recognized for its role as a classical tumor maker, not only as a predictor of lung cancer, but also as a direct correlate of tumor infiltration and metastasis. It has been confirmed that CA125 is associated with lymph node metastasis in lung cancer [50, 51]. CA125 provides important value in judging the extent of lung cancer metastasis and monitoring the progression of lung cancer disease. This study demonstrated the importance of CA125 in determining whether lymph node metastasis is present in lung cancer patients. Surgeons should be more cautious when performing lymph node dissection during lung cancer surgery when faced with patients with high serum CA125 levels.

Mucus is thought to play a key role in the development of cancer, as mucinous adenocarcinoma in many organs is associated with lymph node metastasis and poorer prognosis [52–56]. The mucinous glandular component of the tumor is histologically characterized by cupped and highly columnar epithelial cells and produces mucin, and the mucinous subtype is considered more malignant than other common subtypes of lung adenocarcinoma, such as squamous and alveolar subtypes [57–59]. Some reports with small sample sizes claim a low rate of lymph node metastasis in invasive mucinous adenocarcinoma [60–63]. The results of other studies hold the opposite opinion. The study by Zhu et al. claimed that the mucus subtype is a risk factor for distant metastasis of lung adenocarcinoma [64]. Our findings suggest that the mucus component is one of the risk factors for lymph node metastasis.

Napsin A is a human aspartate protease associated with pepsin, gastrin, renin, and histone protease [65]. IHC studies have demonstrated that Napsin A is expressed in normal human type II lung cells and alveolar macrophages [66]. Strong cytoplasmic staining for napsin A was observed in up to 87% of lung adenocarcinomas [67–71]. In contrast, CK5/6 is a sensitive and relatively specific marker of squamous differentiation [72–74]. The novelty of our study is that for the first time, lymph node metastasis was linked to these two immunohistochemical markers, demonstrating that CK5/6 and napsin A can be used to predict lymph node metastasis in invasive adenocarcinoma. However, the reasons behind why CK5/6 and napsin A can predict lymph node metastasis are still waiting to be explored and studied.

Our study has several advantages compared with other studies. First, for the first time, we included CK5/6, napsin A, and mucus components as influencing factors for lymph node metastasis in our prediction model. Second, the factors in our prediction model are common and easily available in clinical practice. Third, our prediction model has excellent discriminatory power, calibration, and clinical utility. The model is easy to use in clinical practice, and the associated nomogram guides surgeons to quickly select an optimized surgical approach.

Our study has several limitations. First, the analysis was based on retrospective data from a single institution, and the possibility of selection bias cannot be ruled out; results from other centers must be validated. Second, mutation testing was performed according to the patients' wishes. Thus, the sample size for testing their genomics is a subset of the entire cohort, which makes it challenging to include mutation information in a multiple regression analysis. Third, the limited number of cases may lead to potential bias, especially in histological subtype analysis.

## Conclusion

In this study, a clinical prediction model for six risk factors was proposed. For invasive lung cancer, age, SA, CA125, mucin composition, CK5/6, and napsin-A are important risk factors associated with lymph node metastasis. Based on this line chart, surgeons may be able to predict lymph node status before the end of surgery.

### Supplementary Information

**Additional file 1.**

## Data Availability

The data that support the findings of this study are available on request from the corresponding author.

## Abbreviations

LNM( +): Positive for lymph node metastasis
LNM(-): Negative for lymph node metastasis
COPD: Chronic obstructive pulmonary diseases
ASA: American Society of Anesthesiologists
PNI: Prognostic nutritional index
NLR: Neutrophil–lymphocyte ratio
PLR: Platelet-lymphocyte ratio
MLR: Monocyte-lymphocyte ratio
dNLR: Derived neutrophil-to-lymphocyte ratio
NLPR: Neutrophil to lymphocyte and platelet ratio
SIRI: Systemic inflammatory response syndrome
AISI: Aggregate index of systemic inflammation
SII: Systemic inflammation index
LDH: Lactate dehydrogenase
SA: Serum amyloid
5'-NT: 5'-Nucleotidase
Pro-GRP: Pro-gastrin-releasing peptide
SCC: Squamous cell carcinoma
Cyfra21-1: Cytokeratin 19-fragments
CEA: Carcinoembryonic antigen
CA125: Carcinoma antigen 125
NSE: Neuron-specific enolase
BMI: Body mass index
FEV1: Forced expiratory volume in one second
MVV: Maximal voluntary ventilation
CTR: Consolidation-to-tumor ratio
TTF: Thyroid transcription factor 1
PAS: Periodic Acid-Schiff reaction
PAS-D: Periodic Acid-Schiff reaction with diastase
CK 5/6: Cytokeratin 5/6
CK 7: Cytokeratin 7
MUC-AC: Mucin-AC
